# Analysis of characteristics and predictive factors of immune checkpoint inhibitor-related adverse events

**DOI:** 10.20892/j.issn.2095-3941.2021.0052

**Published:** 2021-07-14

**Authors:** Rilan Bai, Naifei Chen, Xiao Chen, Lingyu Li, Wei Song, Wei Li, Yuguang Zhao, Yongfei Zhang, Fujun Han, Zheng Lyu, Jiuwei Cui

**Affiliations:** 1 Cancer Center, the First Hospital of Jilin University, Changchun 130021, China

**Keywords:** Neoplasm, immune checkpoint inhibitors, immune-related adverse events, predictor, efficacy

## Abstract

**Objective:**

We aimed to retrospectively analyze the toxicity profiles and predictors of immune-related adverse events (irAEs) as well as the correlation between irAEs and the clinical efficacy of multi-type immune checkpoint inhibitors (ICIs) in patients with advanced pan-cancer in a real-world setting.

**Methods:**

We retrospectively analyzed data from 105 patients with advanced pan-cancer treated with multi-type ICIs at the First Hospital of Jilin University between January 1, 2016 and August 1, 2020. We used logistic regression analyses to investigate the associations of irAEs with clinical baseline characteristics, blood count parameters, and biochemical indicators during treatment. Receiver operating characteristic curves were used to determine cutoff values for parameters and area under the curve values. Kaplan–Meier and Cox multivariate regression analyses were performed to estimate the relationships of baseline characteristics and irAEs with progression-free survival (PFS) and overall survival (OS).

**Results:**

A lower relative lymphocyte count (cutoff = 28.5%), higher albumin level (cutoff = 39.05 g/L), and higher absolute eosinophil count (AEC) (cutoff = 0.175 × 10^9^/L) were significantly associated with the occurrence of irAEs, among which a higher AEC (cutoff = 0.205 × 10^9^/L) was strongly associated with skin-related irAEs [odds ratios (ORs) = 0.163, *P* = 0.004]. Moreover, a higher lactate dehydrogenase level (cutoff = 237.5 U/L) was an independent predictor of irAEs of grade ≥ 3 (OR = 0.083, *P* = 0.023). In immune cell subgroup analysis, a lower absolute count of CD8^+^CD28^−^ suppressor T cells (OR = 0.806; 95% confidence interval: 0.643–1.011; *P* = 0.062), which are regulatory T lymphocytes, was associated with the occurrence of irAEs, although the difference was not statistically significant. Furthermore, a higher percentage of CD19^+^ B cells was associated with the occurrence of irAEs of grade ≥ 3 (*P* = 0.02) and grade ≥ 2 (*P* = 0.051). In addition, patients with any grade of irAE had a significantly high PFS (8.37 *vs.* 3.77 months, hazard ratios (HR) = 2.02, *P* = 0.0038) and OS (24.77 *vs.* 13.83 months, HR = 1.84; *P* = 0.024).

**Conclusions:**

This retrospective study reports clinical profile data for irAEs in unselected patients in a real-world setting and explored some parameters that may be potential predictive markers of the occurrence, type, or grade of irAEs in clinical practice. Evidence of a correlation between safety and efficacy may facilitate a complete assessment of the risk-benefit ratio for patients treated with ICIs.

## Introduction

Immune checkpoint inhibitors (ICIs), a novel class of antitumor drugs, have shown long-lasting and significant efficacy in the treatment of a variety of malignant tumors by inhibiting immune checkpoints that negatively regulate signaling pathways and activating T lymphocytes to clear tumor cells^1–4^. ICIs currently approved for clinical application include anti-cytotoxic T lymphocyte-associated antigen-4 (CTLA-4) drugs, such as ipilimumab; anti-programmed death 1 (PD-1) drugs, such as nivolumab and pembrolizumab; anti-programmed cell death-ligand 1 (PD-L1) drugs, such as atezolizumab; and a variety of anti-PD-1/PD-L1 drugs made in China, such as toripalimab (JS001), sintilimab (IBI308), and camrelizumab (SHR-1210). These drugs have been continually developed and gradually approved for marketing in China. However, because of the specificity of their targets and mechanisms of action, ICIs may attack normal tissues and organs of the human body while activating the immune system, thereby causing autoimmune and inflammatory effects, known as immune-related adverse events (irAEs), at the corresponding sites^5^. irAEs can affect almost all organs of the human body, most commonly the skin and those belonging to the endocrine, digestive, and respiratory systems^6^. Although irAEs can insidious and variable, most tend to be mild and self-limiting. Severe and even life-threatening irAEs occur in less than 10% of patients^7^; however, the incidence of severe irAEs is as high as 50% with combination immunotherapy^8^. Therefore, with the wide application of ICIs in clinical practice, physicians must fully identify the possible adverse events (AEs) and effective treatment strategies, weigh the benefit-risk ratio, and use drugs rationally to improve the survival outcomes of patients receiving immunotherapy. Although several studies have investigated biomarkers that may predict an increased incidence of irAEs, including T cell repertoire, cytokine levels, and related gene expression patterns, these biomarkers are not extensive or definitive, and are not commonly used in clinical practice. Therefore, no convenient and effective clinical biomarkers are currently available to predict irAEs in patients with advanced tumors. Routine blood testing may be an easy and cost-effective method for detecting irAEs. On the basis of the findings of these tests, in this retrospective cohort study, we aimed to comprehensively analyze the toxicity profiles of irAEs, explore convenient and available markers that can predict irAEs, and elucidate the correlation of irAEs with the clinical efficacy of multi-type ICIs in patients with advanced pan-cancer in a real-world setting.

## Materials and methods

### Patients and indicators

For this retrospective study, the Institutional Review Board of the First Hospital of Jilin University approved the collection of information on all patients with advanced pan-cancer who received ICI therapy in our hospital between January 1, 2016 and August 1, 2020. Patients who withdrew from treatment for reasons other than disease progression or unacceptable toxicity levels after only 1–2 courses of treatment without AE evaluation were excluded. A detailed manual chart review was performed for each patient to record any ICI-related AEs that began after the initiation of treatment, and all patients were followed up for progression and survival until death, loss to follow-up, or withdrawal of consent. irAEs were defined as AEs deemed by the investigator to be associated with immunotherapeutic agents, to have a potential immunological basis, and to require frequent monitoring or potential intervention. To reduce bias, this study focused only on irAEs objectively identifiable by medical professionals, and infusion reactions were not included. The irAEs included dermatological, endocrine, pulmonary, gastrointestinal, hepatic, neurological, hematological, and other rare AEs. Occurrence of only one of these events was defined as a “single-site” irAE, and occurrence of 2 or more events was defined as a “multi-site” irAE. The clinical severity of irAEs was graded according to the Common Terminology Criteria Adverse Events V4.0. Data on characteristics such as age, gender, Eastern Cooperative Oncology Group Performance Status Scale (ECOG PS) score, body mass index (BMI), smoking status, tumor type and stage, distant metastasis, previous treatment, number of lines of treatment, immunotherapy regimens, findings of available laboratory tests [including blood count and related ratio parameters, baseline lactate dehydrogenase (LDH) level, thyroid function indicators, and partially available venous immune cell count], and those of imaging examination, were retrieved from individual medical record review. The primary endpoint of the study was the occurrence of irAEs, and the secondary endpoints were the occurrence of 2 or more irAEs and irAEs of grade ≥ 3 with ICI drug discontinuation. Attending physicians and nurses performed physical examinations and assessed and recorded irAEs every 4 weeks throughout the treatment period. Patients were divided into 2 groups according to the occurrence of irAEs: an irAE group and a non-irAE group. Overall survival (OS) was defined as the time from treatment initiation to death due to any cause, with censoring of patients who were still alive at the date of follow-up. Progression-free survival (PFS) was defined as the time from treatment initiation to disease progression or death from any cause, whichever occurred first. Patients who survived without disease progression were censored at follow-up.

### Statistical analysis

The data are summarized with basic descriptive statistics. The association between categorical variables and events was analyzed with logistic regression: univariate logistic regression analysis was first applied to identify the variables significantly associated with irAEs and to explain the sample size when deriving the model; all variables that were significant at an alpha level less than 0.1 were entered into the multiple logistic regression model to finalize independent correlates of events. A receiver operating characteristic curve was used to calculate cutoff values for the laboratory parameters and the area under the curve^9^. The cutoff point was determined with Youden’s index. The odds ratio (OR) for each cutoff point was calculated by using the results from each of the corresponding logistic regression analyses. The cutoff date for survival analysis was August 1, 2020. Survival probabilities were estimated with Kaplan–Meier curves and log-rank tests, and all covariates with *P* values less than 0.1 were included in a multivariate Cox proportional hazard model to estimate hazard ratios (HRs) and 95% confidence intervals (CIs). Throughout the analyses, all *P* values were based on a 2-sided hypothesis, and those less than 0.05 were considered statistically significant. In assessing the predictor model, the Hosmer-Lemeshow goodness-of-fit test was used to assess the completeness and predictive accuracy of the model. All statistical analyses were performed in GraphPad Prism version 6.0 for Mac and SPSS 22.0.

## Results

### Patient characteristics and treatment

In this retrospective study, 119 patients with advanced tumors (stage IV or stage III tumors that progressed after treatment) who received ICIs were examined; 14 patients who refused treatment for various reasons other than disease progression after 1–2 courses of treatment without AE evaluation were excluded; and 105 patients were included in the final analysis. Among them, 77.14% and 22.86% of patients were men and women, respectively; the mean age was 61 years (range, 24–84 years). All patients had an ECOG score of 0 or 1. The cancer types of the patients were as follows: lung cancer, 52 patients; melanoma, 15 patients; liver cancer, 9 patients; esophageal cancer, 11 patients; urothelial cancer, 8 patients; gastric cancer, 5 patients; and other types of cancers (including hypopharyngeal, nasopharyngeal, colon, and pancreatic cancers and orbital malignancy), 5 patients. Of these patients, 40 and 16 showed metastasis to at least one distant organ and to 2 or more distant organs, respectively. Thirteen patients had bone metastases, and 16 had liver metastases. With regard to previous treatment, 63.81%, 20.95%, 7.62%, 2.86%, and 8.57% of patients received previous chemotherapy, radiotherapy, targeted therapy, immunotherapy, and other treatment regimens (including interferon therapy and intervention/radiofrequency ablation therapy), respectively (**Table 1**). Of the 105 patients, 82 (78.10%) received anti-PD-1 therapy {nivolumab, 16 patients; pembrolizumab, 14 patients; and anti-PD-1 drugs made in China [including toripalimab (JS001), sintilimab (IBI308), tislelizumab (BGB-A317), and camrelizumab (SHR-1210)], 52 patients}; 13 patients (12.38%) received anti-PD-L1 therapy (atezolizumab); and 10 patients (9.52%) received anti-PD-1 combined with anti-CTLA-4 therapy (nivolumab combined with ipilimumab). Among the included patients, 28 (26.67%) were treated with first-line therapy, and 77 (73.33%) were treated with non-first-line therapy.

**Table 1:** Baseline characteristics of the patients

Variable	Number of patients, *n*/value	Percentage (%)	irAE group and non-irAE group	
			*χ^2^*	*P*
Gender			0.044	0.834
Male	81	77.14		
Female	24	22.86		
Age, years			2.308	0.129
Median	61			
Scope	24–84			
ECOG PS			3.137	0.077
0	17	16.19		
1	88	83.81		
BMI			0.054	0.816
Mean value	23.55 ± 0.36 (95% CI 22.83–24.27)			
Scope	15.8–31.2			
Tumor types				
Lung cancer	52	49.52		
Melanoma	15	14.29		
Esophageal cancer	11	10.48		
Liver cancer	9	8.57		
Urothelial carcinoma	8	7.62		
			
Gastric cancer	5	4.76		
Other types of tumors	5	4.76		
Distant metastasis			2.638	0.104
Non-	65	61.90		
One or more-	40	38.10		
Prior therapy				
Chemotherapy	67	63.81		
Radiotherapy	22	20.95		
Targeted therapy	8	7.62		
Immunotherapy	3	2.86		
Other therapies (interferon therapy, interventional/radiofrequency ablation therapy)	9	8.57		
Treatment lines			3.193	0.074
First-line	28	26.67		
Non first-line	77	73.33		
Treatment regimen				
Anti-PD-1	82	78.10		
Anti-PD-L1	13	12.38		
Anti-PD-1 + anti-CTLA-4	10	9.52		

irAEs, immune-related adverse events; PD-1, programmed cell death protein-1; PD-L1, programmed cell death ligand-1; CTLA-4, cytotoxic T-lymphocyte-associated protein-4; ECOG PS, Eastern Cooperative Oncology Group performance status.

### Characteristics and management of irAEs

The incidence of irAEs in the 105 patients with advanced tumors was as follows: any grade, 41 (39.05%); grade 2 or higher, 20 (19%); and grade 3 or higher, 10 (9.5%). Each irAE occurred at different time points in each patient. We found that the time to the first occurrence of irAE was 1.4 months, and most (87.8%) patients experienced the first occurrence of irAEs within 3 months after the initiation of the treatment. The specific details of irAEs were as follows: 19 patients (18.10%) had endocrine AEs, of whom 17 were grade 1–2 AEs (mainly hypothyroidism/hyperthyroidism), and 2 patients had grade 3 AEs (diabetes mellitus); 14 patients (13.33%) had skin responses, all of which were of grade 1–2, with rash being the most common AE; 13 patients (12.38%) had immune-related liver injury, among whom 6 (5.71%) had grade 3–4 AEs or discontinued treatment due to liver injury, which was considered to be associated with anti-PD-1 plus anti-CTLA4 therapy; 6 patients (5.71%) had hematological AEs, and one discontinued the use of drugs, owing to a deficiency of coagulation factor VIII; 4 patients (3.81%) had pancreas-related AEs, of whom 3 had severe irAEs; and among the remaining patients, 3 patients (2.86%) each had grade 2 immune-related pneumonia and grade 1 nerve injury, 2 patients had (1.90%) grade 1 gastrointestinal symptoms, and 11 patients (10.48%) had other AEs, including increased LDH, creatine kinase, and creatine kinase isoenzyme levels, as well as myalgia or back pain, all of which were of low grade (**Table 2**). No irAE-related deaths occurred during the study. Of the 41 patients with irAEs of any grade, 20 had “multi-site” irAEs (number: 2; range, 2–7), and 21 had “single-site” irAEs. The time to onset of the first irAE was 1.37 (0.43–4.87) months for “single-site” irAEs and 1.43 (0.2–10.1) months for “multi-site” irAEs. The most common symptoms of “multi-site” irAEs were endocrine events [65% (13/20)], hepatic events [45% (9/20)], cutaneous events [40% (8/20)], and hematologic events [30% (6/20)]. The most commonly observed combinations were endocrine and cutaneous events [25% (5/20)], endocrine and hematologic events [20% (4/20)], and endocrine and hepatic AEs [20% (4/20)]. The most common “single-site” irAEs were endocrine events [28.57% (6/21)] and cutaneous events [23.81% (5/21)].

**Table 2:** Summary of organ-specific irAEs

Types of irAEs	Grade	Number of patients, *n*	Percentage (%)	irAEs of grade≥ 3, *n*
Endocrine AEs	1–3	19	18.10	2
Skin responses	1–2	14	13.33	0
Immune-related liver injury	1–4	13	12.38	6
Hematologic AEs	1–2	6	5.71	0
Pancreas-related AEs	2–4	4	3.81	3
Immune-related pneumonia	2	3	2.86	0
Nerve injury	1	3	2.86	0
Gastrointestinal symptom	1	2	1.90	0
Immune-related kidney injury	1	1	0.95	0
Immune-related cardiac injury	1	1	0.95	0
Other irAEs	1–2	11	10.48	0

irAEs, immune-related adverse events.

Some differences in the incidence of irAEs according to the tumor types and therapeutic drugs were found, but these differences were not statistically significant. The highest incidence of irAEs was observed in patients with esophageal cancer [81.8% (9/11)]—a finding that may be closely associated with most patients having chosen combination immunotherapy— followed by patients with lung cancer [40.4% (21/52)], urothelial cancer [37.5% (3/8)], liver cancer [33.3% (3/9)], melanoma [20% (3/15)], gastric cancer [20% (1/5)], and other types of cancers [20% (1/5)]. Of the 41 patients with irAEs, 21 (51.2%) had lung cancer; 9 (22.0%) had esophageal cancer; 3 (7.32%) each had melanoma, liver cancer, and urothelial cancer; and one each had gastric and pancreatic cancers. Regarding the safety of different drugs, although the incidence of irAEs of any grade [7.69%, (1/13 patients)] and irAEs of grade ≥ 3 (0 patient) in patients taking anti-PD-L1 drugs was lower than that in those taking anti-PD-1 drugs [39.02% (32/82 patients) and 7.32% (6/82 patients), respectively], the difference was not statistically significant. In particular, the incidence of any-grade irAEs and irAEs of grade ≥ 3 was highest among patients undergoing a double ICI therapy combination [80% (8/10) and 40% (4/10), respectively].

**Figure 1:**
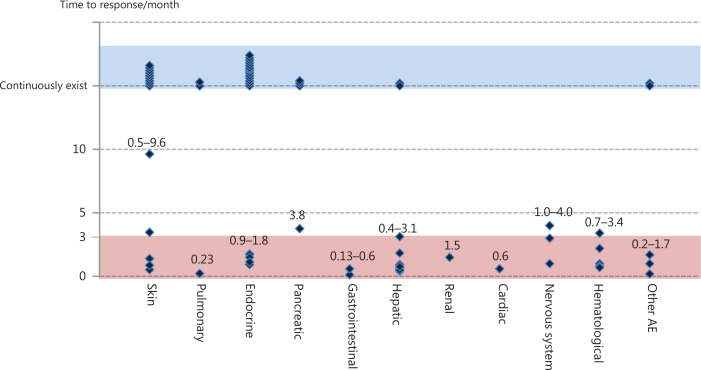
Time to response for each type of irAE. The dark blue diamond-shaped pattern indicates the time to response for each type of irAE for each patient, most of which resolved within 3 months (red areas) after standard treatment; blue areas indicate irAEs that persisted throughout the treatment process or for some time after treatment, most of which were skin- and endocrine-related events. irAEs, immune-related adverse events.

For all patients in our study, irAEs were managed according to the related guidelines^10^. For mild irAEs, drug interruption, symptomatic treatment, and close monitoring were performed as appropriate; all patients with grade 3–4 immune-related liver injury showed improvement after intravenous steroid therapy combined with hepatoprotective, enzyme reduction, and symptomatic and supportive treatment. Patients with grade 4 events permanently discontinued drug use, and some patients with grade 3 events chose to continue drug treatment after the recovery of liver function; for patients with grade 2 pancreatitis and grade 4 lipase elevation, the drug was permanently discontinued, and gradual improvement was observed after intravenous methylprednisolone treatment; one patient developed factor VIII deficiency, which improved after the discontinuation of the drug and supplementation with coagulation factors or transfusion of plasma or cryoprecipitate; grade 2 interstitial pneumonia improved after steroid therapy and immunotherapy were continued; grade 1–2 rash and pruritus were treated with loratadine for anti-allergic therapy; for grade 2–3 endocrine events, such as diabetes and hypothyroidism, drug administration was continued after the administration of hypoglycemic therapy and hormone replacement therapy to maintain stable conditions. No severe irAEs evolved into refractory events. In **Figure 1**, we provide details on the time to response for each type of irAE. Most irAEs completely resolved within 3 months after administration of standard treatment. However, some irAEs persisted throughout the treatment process or for some time after treatment, among which endocrine and skin AEs accounted for a relatively high proportion. Nonetheless, most of the irAEs did not affect the normal progress of immunotherapy.

### Analysis of predictors of irAEs

#### Analysis of predictors in all patients

All patients were divided into irAE and non-irAE groups. There were no significant differences in baseline characteristics (age, gender, ECOG PS score, BMI, distant metastasis, and number of treatment lines) between the groups (*P* > 0.05). The association between the test variables and irAEs was analyzed with univariate and multivariate logistic regression. Univariate analysis showed that the relative lymphocyte count (RLC), albumin (ALB) level, absolute eosinophil count (AEC), platelet (PLT) count, and LDH level were significant factors associated with the occurrence of irAEs. Multivariate analysis confirmed that a lower RLC (cutoff = 28.5%), higher ALB (cutoff = 39.05 g/L), and higher AEC (cutoff = 0.175 × 10^9^/L) were independent factors associated with the occurrence of irAEs (**Table 3**).

**Table 3:** Predictors of irAEs in all patients

Variable	Univariate analysis			Multivariate analysis		
	OR	95% CI	*P*	OR	95% CI	*P*
Gender	1.76	0.66–4.70	0.262			
Age	0.60	0.26–1.35	0.217			
ECOG PS	0.42	0.13–1.30	0.160			
BMI	0.66	0.29–1.48	0.313			
Distant metastasis	0.44	0.19–1.03	0.06			
Treatment lines	2.03	0.84–4.94	0.117			
RLC (cutoff = 28.5%)	3.32	1.28–8.63	0.014*	3.60	1.16–11.19	0.027*
AEC (cutoff = 0.175 × 10^9^/L)	0.32	0.14–0.76	0.010*	0.29	0.10–0.82	0.020*
PLT (cutoff = 232.5 × 10^9^/L)	2.77	1.14–6.73	0.025*			0.122
ALB (cutoff = 39.05 g/L)	0.25	0.10–0.66	0.005**	0.18	0.55–0.58	0.004**
LDH (cutoff = 214.5 U/L)	0.377	0.147–0.967	0.042*			0.139

*P < 0.05; **P < 0.01. irAEs, immune-related adverse events; ECOG PS, Eastern Cooperative Oncology Group performance status; RLC, relative lymphocyte count; AEC, absolute eosinophil count; PLT, platelet; ALB, albumin; LDH, lactate dehydrogenase.

#### Analysis of predictors in the ICI monotherapy group

Given the possible effects of the immune combination therapy regimen on the results of irAE analysis, we analyzed the ICI monotherapy group (95 cases) separately. Univariate analysis showed that RLC, ALB level, AEC, and PLT count were significantly correlated with the occurrence of irAE (*P* < 0.05), whereas other factors, such as age, gender, ECOG PS score, BMI, smoking status, distant metastasis, and number of treatment lines, were not significantly correlated. The results of multivariate analysis in the ICI monotherapy group were consistent with those in the total population; moreover, lower RLC, higher ALB, and higher AEC were found to be independent factors associated with irAEs. Among patients who underwent different treatment regimens (PD-1 or PD-L1), both univariate (*P* = 0.056) and multivariate (*P* = 0.052) analyses showed that the occurrence of irAEs in the PD-1 treatment group tended to be higher than that in the PD-L1 group, but the difference was not statistically significant (**Table 4**).

**Table 4:** Predictors of irAEs in the ICI monotherapy group

Variable	Univariate analysis			Multivariate analysis		
	OR	95% CI	*P*	OR	95% CI	*P*
Gender	2.11	0.74–5.99	0.162			
Age	0.50	0.21–1.19	0.116			
ECOG PS	0.38	0.10–1.43	0.152			
BMI	0.77	0.32–1.86	0.566			
Distant metastasis	0.49	0.20–1.22	0.123			
Treatment lines	1.49	0.53–4.15	0.452			
Treatment regimen (PD-1/PD-L1)	7.68	0.95–61.95	0.056			0.052
RLC (cutoff = 28.5%)	3.78	1.29–11.12	0.016*	5.43	1.55–18.99	0.008**
AEC (cutoff = 0.175 × 10^9^/L)	0.32	0.13–0.80	0.015*	0.28	0.10–0.84	0.023*
PLT (cutoff = 232.5 × 10^9^/L)	2.68	1.01–7.11	0.047*			0.080
ALB (cutoff = 39.05 g/L)	0.29	0.10–0.81	0.019*	0.26	0.09–0.80	0.019*
LDH (cutoff = 214.5 U/L)	0.50	0.19–1.31	0.159			

*P < 0.05; **P < 0.01. ICI, immune checkpoint inhibitor; irAEs, immune-related adverse events; ECOG PS, Eastern Cooperative Oncology Group performance status; RLC, relative lymphocyte count; AEC, absolute eosinophil count; PLT, platelet; ALB, albumin; LDH, lactate dehydrogenase; PD-1, programmed cell death protein-1; PD-L1, programmed cell death ligand-1.

#### Analysis of predictors of irAEs in the immune cell subgroup

Data on the immune cell counts in venous blood were available for only 40 of 105 patients in this study. We found that lower CD8^+^ T cell counts were significantly associated with a higher incidence of irAEs (OR = 0.934; 95% CI 0.885–0.985; *P* = 0.012). Subsequently, we found that total CD8^+^ T cell counts could be further subdivided into 2 groups according to CD28 expression—namely CD28^+^CD8^+^ T cells and CD28^−^CD8^+^ T cells— in 15 patients. Further analysis showed that a lower absolute CD8^+^CD28^−^ T cell count (OR = 0.806; 95% CI 0.643–1.011; *P* = 0.062) was associated with the occurrence of irAEs, although the association was not statistically significant, and none of the other parameters were correlated, including blood counts (**Table 5**). Of note, because of the limitation of the number of patients, the results of blood factor analysis in these patients may not be representative of the results in the overall population.

**Table 5:** Predictors of irAEs in the immune cell subgroup

Variable	Logistics regression analysis		
	OR	95% CI	*P*
CD4^+^ T cell	1.00	1.00–1.00	0.182
CD8^+^ T cell	0.934	0.885–0.985	0.012*
CD28^−^CD8^+^ T cell	0.97	0.89–1.05	0.407
CD28^−^CD8^+^ T cell	0.806	0.643–1.011	0.062
CD19^+^ B cell	0.71	0.20–2.55	0.604
NK cell	1.00	1.00–1.00	0.413
Treg cell	1.00	0.99–1.00	0.12

*P < 0.05.

#### Analysis of the predictors of organ-specific irAEs

In this study, the predictors of organ-specific irAEs were analyzed separately, but no factors associated with endocrine events or liver- and lung-related irAEs were found. Only higher AEC (> 0.205 × 10^9^/L) was found to be an independent factor associated with skin-related irAEs (OR = 0.163, 95% CI 0.048–0.554, *P* = 0.004).

#### Analysis of the predictors of different grades of irAEs

Of the 105 patients, 10 (9.5%) had irAEs of grade ≥ 3. Univariate analysis of blood count parameters showed that only a higher LDH level (cutoff = 237.5 U/L) was an independent factor associated with the occurrence of irAEs of grade ≥ 3 (OR = 0.083, 95% CI 0.01–0.707, *P* = 0.023). Among the 40 patients with immune cell parameters, 5 had irAEs of grade ≥ 3. Only a higher percentage of CD19^+^ B cells (cutoff = 8.565%) (OR = 0.063, 95% CI 0.006–0.651, *P* = 0.02) was associated with the occurrence of irAEs of grade ≥ 3. Of the 105 patients, 20 (19%) experienced irAEs of grade ≥ 2. Only a higher LDH level (cutoff = 214.5 U/L) showed a tendency toward association with the occurrence of irAEs ≥ grade 2 (*P* = 0.08) in all patients. The analysis of immune cell parameters in 40 patients also showed that a high percentage of CD19^+^ B cells (cutoff = 6.605%) showed a tendency toward association with the occurrence of irAEs of grade ≥ 2 (*P* = 0.051).

### Correlation analysis between irAEs and survival outcomes

**Figure 2:**
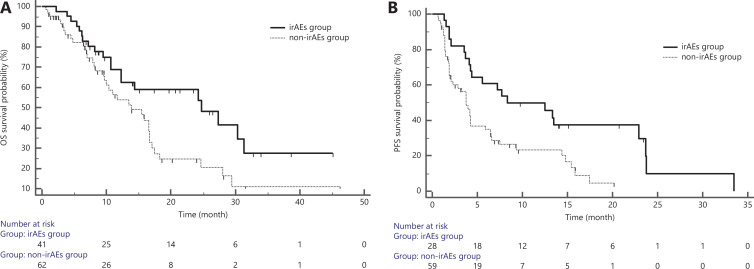
Correlation between irAEs and survival outcomes. A: PFS analysis of the irAE group and non-irAE group; B: OS analysis of the irAE group and non-irAE group. irAEs, immune-related adverse events; PFS, progression-free survival; OS, overall survival.

OS data were available for 103 of the 105 patients, and PFS data were available for 87 patients, owing to disease progression. We examined the effects of baseline characteristics (age, gender, ECOG PS score, BMI, distant metastases, and number of treatment lines) and irAEs on PFS and OS, and performed multivariate analysis when necessary. We found no significant differences in baseline characteristics and efficacy, and only irAEs were independent factors associated with survival outcomes (**Table 6**). A significant difference in PFS was observed between the any-grade irAE group and the non-irAE group [mean PFS: 8.37 months (95% CI 4.37–22.9) *vs.* 3.77 months (95% CI 2.1–4.27), HR = 2.02, 95% CI 1.25–3.26, *P* = 0.0038; 12-month PFS: 43% *vs.* 12%, HR = 0.18, 95% CI 0.06–0.53, *P* = 0.002] (**Figure 2A**). Moreover, the OS significantly differed between the irAE group and non-irAE group [mean OS: 24.77 months (95% CI 10.68–38.86) *vs.* 13.83 months (95% CI 8.16–19.51), HR = 1.84, 95% CI 1.09–3.09, *P* = 0.024; 18-month OS rate: 37% *vs.* 16%, HR = 0.33, 95% CI 0.13–0.84, *P* = 0.020] (**Figure 2B**).

**Table 6:** Correlation analysis between irAEs and survival outcomes

Survival outcomes	Variable	Kaplan–Meier analysis			Cox multivariate regression analysis		
		HR	95% CI	*P*	HR	95% CI	*P*
Progression-free survival	Gender	0.66	0.38–1.13	0.16			
	Age	0.80	0.49–1.29	0.34			
	ECOG PS	0.80	0.41–1.58	0.49			
	BMI	0.59	0.36–0.96	0.030*	0.67	0.40–1.13	0.1371
	Distant metastasis	0.82	0.49–1.36	0.42			
	Treatment lines	1.05	0.58–1.91	0.86			
	irAEs	2.02	1.25–3.26	0.0038**	2.18	1.22–3.90	0.0087**
Overall survival	Gender	0.78	0.41–1.46	0.46			
	Age	0.86	0.51–1.44	0.56			
	ECOG PS	1.04	0.51–2.09	0.92			
	BMI	0.91	0.54–1.54	0.73			
	Distant metastasis	0.69	0.40–1.19	0.16			
	Treatment lines	0.97	0.53–1.79	0.86			
	irAEs	1.84	1.09–3.09	0.024*			

*P < 0.05; **P < 0.01. irAEs, immune-related adverse events; ECOG PS, Eastern Cooperative Oncology Group performance status.

## Discussion

With the widespread use of ICIs in anti-cancer treatment, irAEs have received increasing attention from clinicians and researchers. Although most of the reported common irAEs (namely, rash, thyroid dysfunction, colitis, and diarrhea) are easily managed and treated, some severe or rare irAEs still significantly affect the efficacy and course of immunotherapy in patients, and the occurrence of irAEs in clinical practice is personalized, complex, and unpredictable. Notably, the incidence data and characteristic spectra of irAEs associated with several classes of ICIs (mainly PD-1/PD-L1 antibodies) for different cancer types are similar^11–16^, thus reflecting commonalities in the mechanisms of irAEs occurrence. Specifically, irAEs are mainly associated with abnormal activation of autoimmune T lymphocytes. Therefore, the prediction of the occurrence of irAEs during treatment of pan-cancer with different ICIs is an urgent problem remaining to be solved. However, no prior study has conducted an in-depth exploration of the overall occurrence and predictive markers regarding this event in a real-world setting. Therefore we conducted a retrospective analysis of patients with advanced pan-cancer who were treated with different ICIs to fully assess the incidence and toxicity profiles of irAEs in real clinical practice; to identify the predictors of irAEs from multiple factors (including clinical baseline characteristics, blood count parameters, and biochemical indicators); and to explore the correlation between irAEs and clinical outcomes.

First, the incidence of irAEs of any grade observed in this study was 39.05%, and that of irAEs of grade ≥ 2 was 21.9%. The incidence of irAEs and the overall toxicity profile were consistent with those reported in previous studies focusing on ICI-based treatment^11–13^, which have reported an incidence of irAEs as high as 70% in patients treated with anti-CTLA4 and 50% in those treated with anti-PD-1/PD-L1 antibodies^14–16^. A meta-analysis published in 2015 including 1,265 patients from 22 clinical trials^17^ has reported an overall incidence of 61% for irAEs of any grade and 17% for high-grade irAEs in patients receiving ipilimumab (3 mg/kg). Previous randomized clinical trials have shown that ICIs are generally well tolerated and that the incidence of irAEs of grade ≥ 3 with anti-PD-1/PD-L1 therapy is less than 15%^18^. Our study showed that the incidence of irAEs of grade ≥ 3 was 9.5%, thus indicating that ICIs are relatively safe. All patients with high-grade irAEs in the study showed amelioration of AEs after the discontinuation of ICI treatment and/or administration of treatment with steroids. None of the patients developed refractory AEs, and there were no deaths caused by treatment-related toxicity. Overall, the first median time of the occurrence of irAEs was 1.4 months after the start of ICIs: 1.37 (0.43–4.87) months for “single-site” irAEs and 1.43 (0.2–10.1) months for “multi-site” irAEs. The time (kinetics) of appearance of irAEs is usually determined by the affected organ system^19^, from early onset (1 week) to delayed events (up to 26 weeks for acute kidney injury), with a typical onset window of 4–12 weeks. Most events occur in the first 2-month period, known as the “critical pharmacovigilance window.” In our study, the first irAE occurred in a patient during the 10th treatment course, thus reflecting the delayed response to immunotherapy. A study including 70 patients with advanced non-small cell lung cancer (NSCLC) treated with nivolumab has reported that 28 patients (40%) had irAEs, of which 21 patients (75%) experienced irAEs within 8 weeks, and the median onset time was 5 weeks^20^. The most recent study by Maillet et al.^21^, including 435 patients undergoing ICI treatment with irAEs of grade ≥ 2, has reported that 126 (29%) and 49 patients (11%) had irAEs of grade ≥ 2 and grade ≥ 3, respectively, and the median time to the occurrence of the first irAEs of grades ≥ 2 and 3 was 0.8 (95% CI 0.5–1.8) and 0.6 month (95% CI 0.2–1.4), respectively.

With regard to the incidence of different types of irAEs, a recent systematic review and meta-analysis^22^ including 125 clinical trials involving 20,128 patients has shown that among immune-related endocrine disorders, the most common all-grade AEs are hypothyroidism (6.07%) and hyperthyroidism (2.82%), followed by hyperglycemia (1.20%), thyroiditis (0.75%), and adrenal insufficiency (0.69%). The most common irAEs of all grades are diarrhea (9.47%), elevated aspartate transaminase level (3.39%), vitiligo (3.26%), elevated alanine aminotransferase level (3.14%), pneumonitis (2.79%), and colitis (1.24%). In this study, endocrine disorders and thyroid disorders were the most common AEs. One of these disorders was acute thyroiditis with transient enhancement of thyroid function, followed by conversion to hypothyroidism, and the other was hypothyroidism on regular blood tests; similar results have been reported by Peiro et al.^23^. Patients with endocrine diseases can remain stable after hormone replacement and symptomatic treatment. Although some studies have reported that the presence of anti-thyroid autoantibodies before ICI treatment is a predictor of the occurrence of hypothyroidism^20,24^, and other studies have explored the correlation between the presence of autoantibodies before treatment and the incidence of irAEs, in this study, only indicators of basic thyroid function [thyroid stimulating hormone (TSH), free triiodothyronine (T3), and free thyroxine] were measured, because of an inability to obtain a sufficient number of patients with baseline autoantibody levels. We confirmed that low T3 levels before immunotherapy were significantly associated with the occurrence of irAEs of any grade, and high TSH levels were significantly associated with the occurrence of irAEs of grade ≥ 2. Immune-mediated skin-related disorders, mainly rash with dry and itchy skin, were treated with moisturizers, oral antihistamines, and topical creams; no severe grade events were observed. In this study, the proportion of patients with immune-related liver injury (12.38%, 13 patients) was relatively large; this finding was considered to be associated with the combination of anti-PD-1 and anti-CTLA4 drugs. Although 7 of the 13 patients had severe events, all showed improvement after the initiation of intravenous corticosteroid therapy, and some patients continued to use the drug. In addition, we identified some rare types of irAEs. We observed neurologic events, such as mild numbness of the feet and toes, and facial neuritis (one patient each), as well as hematological events, such as prolonged activated partial thromboplastin time and coagulation factor deficiency, leading to drug withdrawal and reporting serious AE (one patient). Notably, a patient with stage IV gastric differentiated adenocarcinoma successively developed severe systemic AEs 1 week after the administration of nivolumab combined with ipilimumab; the symptoms included dry mouth/dry eyes, grade 2 pneumonia, grade 1 hypothyroidism, grade 4 elevation of lipase level, grade 2 pancreatitis, grade 4 hepatic injury, grade 1 facial neuritis, and grade 2 myalgia. The patient discontinued therapy after 4 courses of treatment because of intolerable severe adverse reactions, although stable disease was evaluated after 2 courses, thus reflecting the individual uniqueness and unpredictability of the immune response. Although the findings of our study differed from those in previous studies in terms of the occurrence of irAEs in each category, because of the limited number of patients in our study, the overall incidence and toxicity profile of irAEs were similar. Patient inclusion criteria in clinical trials are stringent and may result in some rare and unique cases being ignored. In contrast, we report the clinical characteristics of patients in a real-world setting, albeit with limited sample sizes. Larger retrospective surveys are expected to be conducted in a real-world setting in the future to comprehensively characterize the toxicity profiles of irAEs in patients treated with ICIs.

Given that multiple irAEs occur before or close to routine response evaluation, early identification of irAEs is important to optimize the therapeutic benefits of ICIs. As mentioned previously, the data on the incidence and range of irAEs associated with ICIs for different cancer types are similar. However, because the number of patients using each ICI in our study varied significantly, and the sample sizes of some subgroups were very small, a detailed comparison of the incidence of irAEs between drugs could not be made. Overall, we confirmed the absence of statistically significant differences in the occurrence of irAEs between anti-PD-1 and anti-PD-L1 drugs; however, the incidence of toxic events was significantly higher in the combination group. Although several studies have reported biomarkers, such as T cell repertoire, interleukin (IL)-6 levels, and IL-17 levels, that may predict the occurrence of irAEs, complete blood counts have recently been proposed to serve as markers of cancer inflammation and the adaptive immune response. Routine blood testing may be an accessible, easy and cost-effective method to detect irAEs, although its predictive ability has not been elucidated. Therefore, this study examined the predictive ability of baseline peripheral blood counts for irAEs. We found that a lower RLC (cutoff = 28.5%), higher ALB level (cutoff = 39.05 g/L), and higher AEC (cutoff = 0.175 × 10^9^/L) were significantly associated with the occurrence of irAEs of any grade, whereas no significant associations were found between clinical characteristics and irAEs. Several previous studies have shown that cells in the peripheral blood can not only predict tumor response but also indicate the occurrence of irAEs mediated by ICIs. Multivariate analysis by Diehl et al.^25^ has shown that the absolute number of lymphocytes and eosinophils at baseline and 1 month after initial treatment are independent factors significantly associated with a higher incidence of irAEs of grade ≥ 2 in patients with solid tumors (including melanoma, renal cell carcinoma, and urothelial carcinoma) treated with anti-PD-1 antibodies. Schindler et al.^26^ have reported a higher incidence of irAEs in patients with a higher eosinophil count at weeks 4 and 7, and in patients with melanoma treated with ipilimumab who show a change in eosinophil count from baseline. Univariate analysis of the characteristics of patients with melanoma treated with nivolumab has shown that both increased total white blood cell count and decreased RLC, relative to the baseline counts, are associated with severe irAEs of grade ≥ 3, although multivariate analysis has not shown independent correlation^27^. Increased neutrophils may represent a systemic inflammatory response, whereas decreased lymphocyte counts may reflect an impaired cell-mediated specific immune response^28^, thus potentially partly explaining the observations in this study. In addition, our findings might be explained by the redistribution of systemic blood cells, in which substantial infiltration of lymphocytes in the involved organs eventually results in a decrease in circulating blood lymphocytes and an increase in neutrophils. However, the current study does not clearly support this possibility, and the intrinsic mechanisms are worthy of in depth exploration in future studies to better interpret the correlation between circulating blood cells and the occurrence of irAEs.

Furthermore, the correlation between blood cell counts and irAEs in various organs is poorly understood. In this study, the predictors of irAEs at different sites were analyzed separately, and only a higher baseline AEC was found to have a strong association with the occurrence of skin-related irAEs (OR = 0.163, *P* = 0.004). The cutoff value of AEC was determined to be 0.205 × 10^9^/L through receiver operating characteristic curve analysis. A higher baseline AEC revealed by blood tests may be a potential predictor of skin-related AEs. Previous studies have shown an increase in the number of circulating eosinophils and in the infiltration of eosinophils in the dermis in patients who develop cutaneous irAEs while receiving anti-CTLA-4 therapy^29^, and a significant correlation between baseline absolute and relative eosinophil counts and the occurrence of endocrine irAEs at 1 month in patients with melanoma treated with anti-PD-1 antibodies^30^. A recent study has found that higher peripheral blood AECs (≥ 0.125 × 10^9^/L) are associated with greater incidence of ICI-related pneumonitis^31^. Therefore, eosinophils may play an important role in the pathogenesis of anti-PD-1-induced irAEs and may be organ specific; however, the underlying biological mechanism remains to be fully elucidated. Eosinophils have been proposed to function as regulatory cells or effector cells modulating a variety of immune functions, such as activating T cells and attracting tumor-specific CD8^+^ T cells by exerting antigen-presenting functions^32–34^. Results from animal models have also indicated that eosinophils are involved in the regulation of T cell responses^35^. Therefore, eosinophils may be hypothesized to ultimately mediate the inflammatory response by increasing the infiltration of activated T cells.

In the immune cell subgroup, we first preliminarily analyzed the correlation between immune cells and irAEs in 40 patients and found that lower CD8^+^ T cell counts were significantly associated with a higher incidence of irAEs; however, this finding was not consistent with irAEs occurring because of the activation ofthe immune response in normal tissues of the body. Interestingly, after further subdividing total CD8^+^ T cells into 2 groups according to CD28 expression, namely CD28^+^CD8^+^ T cells and CD28^−^CD8^+^ T cells, in 15 patients, we further confirmed that a lower absolute CD28^−^CD8^+^ T cell count (*P* = 0.062) was associated with the occurrence of irAEs, although the difference was not statistically significant. CD8^+^CD28^−^ suppressor T cells are derived from the monoclonal expansion of T cells^36^, which can act directly on antigen-presenting cells, thus downregulating the expression of costimulatory molecules and upregulating inhibitory receptors^37^. In *in vitro* experiments, CD8^+^CD28^−^ T cells inhibited the proliferation of effector CD4^+^ T cells and their secretion of interferon-γ. CD8^+^CD28^−^ T cells negatively modulate the immune response through multiple mechanisms, such as direct negative regulation of adaptive immune responses or limiting the diversity of adaptive immune responses. CD8^+^CD28^−^ T regulatory lymphocytes are usually persistent and functional in human tumors, and inhibit both T cell proliferation and cytotoxicity in a pathogenically relevant manner^38^. CD8^+^CD28^−^ T cells have been shown to suppress experimental inflammatory bowel disease in mice, and repeated stimulation of human peripheral blood lymphocytes with allogeneic APCs *in vitro* causes a loss of lymphocyte proliferative activity, owing to the actions of CD8^+^CD28^−^ regulatory T cells^39^. CD8^+^CD28^−^ T cells are associated with poor response to antitumor therapy and poor prognosis in a variety of tumors. One study has shown that elevated peripheral blood CD8^+^CD28^−^ T cell counts are associated with poor prognosis in metastatic breast cancer, particularly in patients with a high risk of progression receiving first-line chemotherapy, and a higher risk of death than that in patients receiving second-line chemotherapy^40^. In patients with lung cancer, CD8^+^CD28^−^ T cells have elevated Foxp3 expression and show immunomodulatory effects^41^. Moreover, high numbers of CD8^+^CD28^−^ T cells are found in patients with advanced NSCLC, and a decrease in the number of CD8^+^CD28^−^ T cells is correlated with favorable prognosis in tumor management^42^. Recently, several studies have suggested that downregulation of the costimulatory molecule CD28 serves as a marker of senescent T cells; have proposed the concept of a CD8^+^CD28^−^ senescent T population; and have observed an increase in this population in a variety of solid and hematogenous tumors^43–44^. The circulating T cell senescence immune phenotype (CD28^−^CD57^+^KLRG1^+^) has been observed in 28% of patients with advanced NSCLC and is associated with a significantly poorer objective response rate, PFS, and OS after ICI treatment^43^. Senescent T cells induced by CD8^+^CD28^−^ Treg cells have potent regulatory activities and augment the immunosuppression of the tumor microenvironment. Therefore, CD8^+^CD28^−^ T cells are important mediators of Treg-mediated immunosuppression. Blocking Treg-induced senescence of responding immune cells is essential for controlling tumor immunosuppression and restoring effector T cell function. Thus, CD8^+^CD28^−^ T cells contribute to tumor immunosuppression and immunotherapy resistance. Further characterization and study of CD8^+^CD28^−^ T cells should provide new targets for effective immunotherapy and successful cancer control.

However, the role of the immunosuppressive function of CD8^+^ regulatory T cells in immune-mediated AEs has not been investigated. This study showed that patients with relatively higher counts of CD8^+^CD28^−^ suppressor T cells were less likely to have irAEs, possibly because of the inhibitory effect of CD8^+^CD28^−^ suppressor T cells on effector T cells. Increased numbers of CD8^+^CD28^−^ suppressor T cells may represent suppressed and impaired immunity. After the application of ICIs, the systemic immune system is not easily activated to produce a response, including immune-mediated autoimmune and inflammatory effects in normal tissues and organs of the human body. However, the specific mechanism of action of these special cells in irAEs must be further explored through basic experimental studies in the future. Unfortunately, our study had a small sample size, and the difference in the incidence of irAEs between groups was not statistically significant. Nevertheless, to our knowledge, our study is the first to discover the role of CD8^+^CD28^−^ T cells in immune responses and AEs, and these data with a trend toward significance were determined from only a simple peripheral blood examination. Moreover, previous studies have only detected and analyzed CD8^+^ T cells, whereas our study suggests that these cells can be further divided into CD28^+^ and CD28^+^ T cells in subsequent studies to better understand their role in the body’s immune response and the incidence of irAEs. In the future, the sample size must be increased to test the role of CD8^+^CD28^−^ T cells in large-scale prospective studies.

Severe irAEs can significantly affect treatment response or lead to treatment discontinuation; thus, identifying predictors of severe irAEs is essential for the clinical management of patient survival outcomes. This study found that the LDH level (cutoff = 237.5 U/L) was significantly elevated in the group with irAEs of grade ≥ 3 (OR = 0.083, *P* = 0.023). LDH has attracted attention as an indicator of systemic inflammation in recent years. Several studies on various cancer types have shown that high LDH levels are a poor prognostic factor for PFS or OS^45,46^, but their association with the occurrence of irAEs had not been reported. Our study confirms that patients with higher LDH levels may have a higher systemic inflammatory status, which in turn may promote the occurrence of higher grade irAEs. Therefore, the detection of LDH levels in clinical practice may provide a reference for analyzing the severity of irAEs. In addition, we analyzed peripheral blood immune cell counts in patients with different grades of irAEs. Drugs targeting the PD-1/PD-L1 axis can not only trigger potential autoimmune responses by enhancing T cell-mediated mechanisms but also induce autoantibody production *via* B cell-mediated mechanisms, thus further enhancing humoral immune responses, disrupting self-tolerance, and ultimately causing autoimmune diseases by attacking autoantigens^24^. For example, a study has shown that in patients treated with anti-PD-1, the presence of anti-thyroid antibodies at baseline or after the initiation of treatment may trigger immune-related thyroid disease^47^. The autoantibody data in our patients were not analyzed because they were available for only a small number of patients. However, we considered that irAEs may occur because immunotherapy activates B cells and makes the body more sensitive to antigen recognition, thus ultimately resulting in self-damage caused by the immune response *via* autoantibodies. Therefore, in this study, we analyzed small samples of peripheral blood for immune cells. A high percentage of CD19^+^ B cells was associated with the occurrence of irAEs of grade ≥ 3 (OR = 0.063, *P* = 0.02) and grade ≥ 2 (*P* = 0.051), although the latter showed only a trend to significance. Our study further confirmed the cellular and humoral immune mechanisms underlying the development of irAEs, thereby providing important insights into the immunobiology of ICIs and autoimmunity in general. Overall, in exploring the predictors of irAEs, although some indicators have been shown to be closely associated with the occurrence of irAEs, clinicians should always keep in mind that the occurrence of immune-related toxicity may be difficult to predict and diagnose, and may be fatal; this aspect is particularly important in the context of the widespread use of immunotherapy.

Finally, given the similarity between the immunological basis of immune-related toxicity and the clinical benefits of ICIs, several recent retrospective studies have investigated the correlation between irAEs and the efficacy of ICIs, particularly in lung cancer and melanoma, and have shown positive results^20,48–50^. A meta-analysis of 4 prospective studies in different cancers has shown that irAEs of any grade are associated with a high overall response rate but not with PFS^11^. However, Mori et al.^46^ have found a significantly shorter PFS (3 *vs.* 10 months, HR = 2.2, *P* = 0.016) in patients without any-grade irAEs in a study on 153 patients treated with anti-PD-1/PD-L1, although the decrease in mean OS was not significant. Therefore, whether the occurrence of irAEs might reflect treatment response and translate into better survival outcomes in clinical practice was unknown. Consequently, we conducted a retrospective analysis of data on 105 real-world patients, further emphasizing the importance of irAEs in predicting survival outcomes. The overall results showed that patients with any-grade irAEs had significantly better PFS and OS, thus indicating a strong association between the occurrence of irAEs and good treatment efficacy. Our findings initially confirmed that the occurrence of irAEs may translate into better clinical survival benefits, emphasizing that the maintenance of ICI treatment should be a priority in patients with irAEs and that careful management of treatment-related toxicity can maximize the clinical benefits of immunotherapy in patients.

This is a small, retrospective, non-randomized study conducted in a single center with certain limitations; therefore, the results should be interpreted with caution. First, the sample size was small and may have been affected by the intrinsic selection bias associated with the retrospective study design. Second, the definition criteria for irAEs vary among studies, and the determination of irAEs in clinical practice is also subjective; some are diagnosed only by exclusion. Therefore, in the absence of specific descriptions in the medical literature, irAEs of grade 1–2 may be underestimated or overlooked, but the most severe irAEs are less likely to be missed. Third, because patients with long periods of exposure to immunotherapy are more likely to develop immunotoxicity, the duration/observation time of immunotherapy may also be a potential confounding factor. However, 30 of the 41 patients (73.17%) who showed irAEs in this study developed symptoms 8 weeks after treatment, thereby indicating that the risk of AEs is unlikely to increase with longer treatment periods, and AEs are likely to be caused by the abnormal activation of the immune system. Fourth, the patients in this study had different tumor types, and a background of different types and dosage regimens of ICI drugs. Although the spectrum of use of different ICIs in different cancers was similar, confounding factors remained inevitable. Fifth, other studies have reported some irAE-associated biomarkers based on germline genetic factors, indicators of immune repertoire, and cytokine levels (that is, clonality of the T cell repertoire, IL-6 levels, IL-17 levels, human leucocyte antigen typing, and gene polymorphisms), which may more accurately predict AEs and guide clinical decisions. Analysis targeting key cytokines should also be helpful for the prediction and mechanistic exploration of irAEs. For instance, integrated analysis of multiple circulating cytokine analyses may predict irAEs. One study has integrated 11 cytokines that are significantly elevated in patients with severe irAEs at baseline and early during treatment into a toxicity score called the CYTOX score. The CYTOX single toxicity score has been validated as a predictive marker for severe ICI-associated irAEs in patients with melanoma, thus suggesting that a single cytokine toxicity score might potentially contribute to the early management of severe or potentially life-threatening immune-related toxicity^51^. However, because of the lack of tissue samples in most patients, we were unable to assess these biomarkers in our study, and we mainly explored the clinical characteristics and baseline blood biochemical indicators of the patients. Nevertheless, these simple and easily available markers may provide a faster and more convenient method for clinical practice—a possibility that must be further validated to promote the development of the field.

## Conclusions

Overall, this retrospective study reported the clinical profile data of irAEs of unselected patients in a real-world setting and explored several parameters that may potentially serve as conventionally adopted, cost-effective, markers predicting the occurrence, type, or grade of irAEs in clinical practice, thus providing great clinical value. Evidence of a correlation between safety and efficacy may ultimately be integrated into treatment decisions to fully assess the risk-benefit ratio for patients according to the type and severity of irAEs as well as the response. Although these findings require further validation in larger retrospective or prospective randomized controlled studies, we provide important information and reference values for future studies on the predictors of irAEs and their correlation with clinical outcomes.

## References

[1] Goldberg SB, Schalper KA, Gettinger SN, Mahajan A, Herbst RS, Chiang AC (2020). Pembrolizumab for management of patients with NSCLC and brain metastases: long-term results and biomarker analysis from a non-randomised, open-label, phase 2 trial. Lancet Oncol.

[2] Schmid P, Rugo HS, Adams S, Schneeweiss A, Barrios CH, Iwata H (2020). Atezolizumab plus nab-paclitaxel as first-line treatment for unresectable, locally advanced or metastatic triple-negative breast cancer (IMpassion130): updated efficacy results from a randomised, double-blind, placebo-controlled, phase 3 trial. Lancet Oncol.

[3] Larkin J, Chiarion-Sileni V, Gonzalez R, Grob JJ, Rutkowski P, Lao CD (2019). Five-year survival with combined nivolumab and ipilimumab in advanced melanoma. N Engl J Med.

[4] Naumann RW, Hollebecque A, Meyer T, Devlin MJ, Oaknin A, Kerger J (2019). Safety and efficacy of nivolumab monotherapy in recurrent or metastatic cervical, vaginal, or vulvar carcinoma: results from the phase I/II checkmate 358 trial. J Clin Oncol.

[5] Bajwa R, Cheema A, Khan T, Amirpour A, Paul A, Chaughtai S (2019). Adverse effects of immune checkpoint inhibitors (programmed death-1 inhibitors and cytotoxic t-lymphocyte-associated protein-4 inhibitors): results of a retrospective study. J Clin Med Res.

[6] Wang DY, Salem JE, Cohen JV, Chandra S, Menzer C, Ye F (2018). Fatal toxic effects associated with immune checkpoint inhibitors: a systematic review and meta-analysis. JAMA Oncol.

[7] Heinzerling L, Goldinger SM (2017). A review of serious adverse effects under treatment with checkpoint inhibitors. Curr Opin Oncol.

[8] Antonia SJ, Lopez-Martin JA, Bendell J, Ott PA, Taylor M, Eder JP (2016). Nivolumab alone and nivolumab plus ipilimumab in recurrent small-cell lung cancer (checkmate 032): a multicentre, open-label, phase 1/2 trial. Lancet Oncol.

[9] Youden WJ (1950). Index for rating diagnostic tests. Cancer.

[10] Brahmer JR, Lacchetti C, Schneider BJ, Atkins MB, Brassil KJ, Caterino JM (2018). Management of immune-related adverse events in patients treated with immune checkpoint inhibitor therapy: American Society of Clinical Oncology clinical practice guideline. J Clin Oncol.

[11] Weber JS, Hodi FS, Wolchok JD, Topalian SL, Schadendorf D, Larkin J (2017). Safety profile of nivolumab monotherapy: a pooled analysis of patients with advanced melanoma. J Clin Oncol.

[12] Weber JS, D’Angelo SP, Minor D, Hodi FS, Gutzmer R, Neyns B (2015). Nivolumab versus chemotherapy in patients with advanced melanoma who progressed after anti-CTLA-4 treatment (checkmate 037): a randomised, controlled, open-label, phase 3 trial. Lancet Oncol.

[13] Robert C, Schachter J, Long GV, Arance A, Grob JJ, Mortier L (2015). Pembrolizumab versus ipilimumab in advanced melanoma. N Engl J Med.

[14] Brahmer JR, Tykodi SS, Chow LQ, Hwu WJ, Topalian SL, Hwu P (2012). Safety and activity of anti-PD-L1 antibody in patients with advanced cancer. N Engl J Med.

[15] Topalian SL, Hodi FS, Brahmer JR, Gettinger SN, Smith DC, McDermott DF (2012). Safety, activity, and immune correlates of anti-PD-1 antibody in cancer. N Engl J Med.

[16] Devaud C, John LB, Westwood JA, Darcy PK, Kershaw MH (2013). Immune modulation of the tumor microenvironment for enhancing cancer immunotherapy. Oncoimmunology.

[17] Brahmer JR, Tykodi SS, Chow LQM, Hwu WJ, Topalian SL, Hwu P (2012). Safety and activity of anti–PD-L1 antibody in patients with advanced cancer. N Engl J Med.

[18] Bertrand A, Kostine M, Barnetche T, Truchetet ME, Schaeverbeke T (2015). Immune related adverse events associated with anti-CTLA-4 antibodies: systematic review and meta-analysis. BMC Med.

[19] Martins F, Sofiya L, Sykiotis GP, Lamine F, Maillard M, Fraga M (2019). Adverse effects of immune-checkpoint inhibitors: epidemiology, management and surveillance. Nat Rev Clin Oncol.

[20] Toi Y, Sugawara S, Kawashima Y, Aiba T, Kawana S, Saito R (2018). Association of immune-related adverse events with clinical benefit in patients with advanced non-small-cell lung cancer treated with nivolumab. Oncologist.

[21] Maillet D, Corbaux P, Stelmes JJ, Dalle S, Locatelli-Sanchez M, Perier-Muzet M (2020). Association between immune-related adverse events and long-term survival outcomes in patients treated with immune checkpoint inhibitors. Eur J Cancer.

[22] Wang Y, Zhou S, Yang F, Qi X, Wang X, Guan X (2019). Treatment-related adverse events of PD-1 and PD-L1 inhibitors in clinical trials: a systematic review and meta-analysis. JAMA Oncol.

[23] Peiró I, Palmero R, Iglesias P, Díez JJ, Simó-Servat A, Marín JA (2019). Thyroid dysfunction induced by nivolumab: searching for disease patterns and outcomes. Endocrine.

[24] Toi Y, Sugawara S, Sugisaka J, Ono H, Kawashima Y, Aiba T (2019). Profiling preexisting antibodies in patients treated with Anti-PD-1 therapy for advanced non-small cell lung cancer. JAMA Oncol.

[25] Diehl A, Yarchoan M, Hopkins A, Jaffee E, Grossman SA (2017). Relationships between lymphocyte counts and treatment-related toxicities and clinical responses in patients with solid tumors treated with PD-1 checkpoint inhibitors. Oncotarget.

[26] Schindler K, Harmankaya K, Kuk D (2014). Correlation of absolute and relative eosinophil counts with immune-related adverse events in melanoma patients treated with ipilimumab. Int J Obes Relat Metab Disord.

[27] Shahabi V, Berman D, Chasalow SD, Wang L, Tsuchihashi Z, Hu B (2013). Gene expression profiling of whole blood in ipilimumab-treated patients for identification of potential biomarkers of immune-related gastrointestinal adverse events. J Transl Med.

[28] Grivennikov SI, Greten FR, Karin M (2010). Immunity, inflammation, and cancer. Cell.

[29] Jaber SH, Cowen EW, Haworth LR, Booher SL, Berman DM, Rosenberg SA (2006). Skin reactions in a subset of patients with stage IV melanoma treated with anti-cytotoxic T-lymphocyte antigen 4 monoclonal antibody as a single agent. Arch Dermatol.

[30] Nakamura Y, Tanaka R, Maruyama H, Ishitsuka Y, Okiyama N, Watanabe R (2019). Correlation between blood cell count and outcome of melanoma patients treated with anti-PD-1 antibodies. Jpn J Clin Oncol.

[31] Chu X, Zhao J, Zhou J, Zhou F, Jiang T, Jiang S (2020). Association of baseline peripheral-blood eosinophil count with immune checkpoint inhibitor-related pneumonitis and clinical outcomes in patients with non-small cell lung cancer receiving immune checkpoint inhibitors. Lung Cancer.

[32] Carretero R, Sektioglu IM, Garbi N, Salgado OC, Beckhove P, Hämmerling GJ (2015). Eosinophils orchestrate cancer rejection by normalizing tumor vessels and enhancing infiltration of CD8(+) T cells. Nat Immunol.

[33] Simon SCS, Utikal J, Umansky V (2019). Opposing roles of eosinophils in cancer. Cancer Immunol Immunother.

[34] Wen T, Rothenberg ME (2016). The regulatory function of eosinophils. Microbiol Spectr.

[35] Jacobsen EA, Ochkur SI, Pero RS, Taranova AG, Protheroe CA, Colbert DC (2008). Allergic pulmonary inflammation in mice is dependent on eosinophil-induced recruitment of effector T cells. J Exp Med.

[36] Chiu WK, Fann M, Weng NP (2006). Generation and growth of CD28nullCD8+ memory T cells mediated by IL-15 and its induced cytokines. J Immunol.

[37] Chang CC, Ciubotariu R, Manavalan JS, Yuan J, Colovai AI, Piazza F (2002). Tolerization of dendritic cells by T(S) cells: the crucial role of inhibitory receptors ILT3 and ILT4. Nat Immunol.

[38] Filaci G, Fenoglio D, Fravega M, Ansaldo G, Borgonovo G, Traverso P (2007). CD8+ CD28-T regulatory lymphocytes inhibiting T cell proliferative and cytotoxic functions infiltrate human cancers. J Immunol.

[39] Song Q, Ren J, Zhou X, Wang X, Song G, Hobeika A (2018). Circulating CD8(+)CD28(-) suppressor T cells tied to poorer prognosis among metastatic breast cancer patients receiving adoptive T-cell therapy: a cohort study. Cytotherapy.

[40] Vlad G, Cortesini R, Suciu-Foca N (2005). License to heal: bidirectional interaction of antigen-specific regulatory T cells and tolerogenic APC. J Immunol.

[41] Meloni F, Morosini M, Solari N, Passadore I, Nascimbene C, Novo M (2006). Foxp3 expressing CD4+ CD25+ and CD8+CD28-T regulatory cells in the peripheral blood of patients with lung cancer and pleural mesothelioma. Hum Immunol.

[42] Chen C, Chen D, Zhang Y, Chen Z, Zhu W, Zhang B (2014). Changes of CD4+CD25+FOXP3+ and CD8+CD28-regulatory T cells in non-small cell lung cancer patients undergoing surgery. Int Immunopharmacol.

[43] Ferrara R, Naigeon M, Auclin E, Duchemann B (2021). Circulating T-cell immunosenescence in patients with advanced non-small cell lung cancer treated with single-agent PD-1/PD-L1 Inhibitors or platinum-based chemotherapy. Clin Cancer Res.

[44] Huff WX, Kwon JH, Henriquez M, Fetcko K, Dey M (2019). The evolving role of CD8(+)CD28(-) immunosenescent t cells in cancer immunology. Int J Mol Sci.

[45] Zhang Z, Li Y, Yan X, Song Q, Wang G, Hu Y (2019). Pretreatment lactate dehydrogenase may predict outcome of advanced non small-cell lung cancer patients treated with immune checkpoint inhibitors: a meta-analysis. Cancer Med,.

[46] Mori K, Kimura S, Parizi MK, Enikeev DV, Shariat SF (2019). Prognostic value of lactate dehydrogenase in metastatic prostate cancer: a systematic review and meta-analysis. Clin Genitourin Cancer.

[47] Kobayashi T, Iwama S, Yasuda Y, Okada N, Tsunekawa T, Onoue T (2018). Patients with antithyroid antibodies are prone to develop destructive thyroiditis by nivolumab: a prospective study. J Endocr Soc.

[48] Rzepecki AK, Cheng H, McLellan BN (2018). Cutaneous toxicity as a predictive biomarker for clinical outcome in patients receiving anticancer therapy. J Am Acad Dermatol.

[49] Nakamura Y, Tanaka R, Asami Y, Teramoto Y, Imamura T, Sato S (2017). Correlation between vitiligo occurrence and clinical benefit in advanced melanoma patients treated with nivolumab: a multi-institutional retrospective study. J Dermatol.

[50] Rogado J, Sánchez-Torres JM, Romero-Laorden N, Ballesteros AI, Pacheco-Barcia V, Ramos-Leví A (2019). Immune-related adverse events predict the therapeutic efficacy of anti-PD-1 antibodies in cancer patients. Eur J Cancer.

[51] Lim SY, Lee JH (2019). Circulating Cytokines Predict Immune-related toxicity in melanoma patients receiving anti-PD-1-based immunotherapy. Clin Cancer Res.

